# 95% effective volume of ropivacaine for ultrasound‑guided supra‑inguinal fascia iliaca compartment block

**DOI:** 10.1186/s12871-023-02049-5

**Published:** 2023-03-29

**Authors:** Can Zhang, Wei Dai, Kaihua He

**Affiliations:** anesthesiology department, The First Affiliated Hospital of Chongqing Medical University, Chongqing, China

**Keywords:** Supra-inguinal fascia iliaca compartment block, 95% effective volume, Ultrasound, Ropivacaine, Biased-coin design

## Abstract

**Background:**

Hip arthroplasty is effective in treating hip diseases, such as osteoarthritis and hip fracture, but it often brings severe trauma and pain. In recent years, ultrasound-guided supra-inguinal fascia iliaca compartment block(S-FICB) has become a widely used nerve block method for analgesia in hip arthroplasty.

**Methods:**

Fifty-three patients preparing for hip arthroplasty were prospectively enrolled. S-FICB was performed under ultrasound guidance, and inject 0.33% ropivacaine into the space. Using the biased-coin design (BCD) sequential allocation method. The initial volume of 0.33% ropivacaine was 30ml. In case of failure, the next patient received a higher volume (defined as the previous volume with an increment of 1.2 mL). If the previous patient had a successful block, the next patient was randomized to a lower volume (defined as the previous volume with a decrement of 1.2 mL), with a probability of b = 0.05, or the same volume, with a probability of 1 − b = 0.95. The study was stopped when 45 successful blocks were achieved.

**Results:**

Forty-five patients (84.9%) were blocked successfully. The 95% effective volume (EV95) was 34.06ml (95%CI 33.35 ~ 36.28ml). There were 31 patients with non-fracture in this study. The quadriceps muscle strength decreased in only two patients. Moreover, they both received 34.8ml of ropivacaine for S-FICB. Twenty-two patients had hip fractures. There were 3 patients (14%) with failed blocks and 19 patients (86%) with successful blocks. However, all fracture patients experienced less pain after S-FICB.

**Conclusion:**

EV95 of 0.33% ropivacaine for ultrasound-guided S-FICB was 34.06ml.

**Trial registration:**

The trial was registered at the Chinese Clinical Trial Registry (registration number: ChiCTR2100052214, registration date: 2021 October 22).

## Introduction

Hip arthroplasty has become an essential method for treating hip fractures, osteonecrosis of the femoral head, and other diseases. The operation involves a wide range of tissues, and most patients have severe pain after the operation. Therefore, effective perioperative analgesia is helpful for patients to carry out the early functional exercise, reduce bed rest time, and reduce the risk of adverse events such as lower limb deep venous thrombosis and pulmonary embolism. In recent years, ultrasound-guided peripheral nerve block has become an important part of multimodal analgesia. Ultrasound-guided S-FICB has become a widely used nerve block method for analgesia in hip arthroplasty. Ultrasound-guided S-FICB can not only effectively spread local anesthetic (LA) in the fascia iliaca compartment (FIC) but also improve the success of the block of the femoral nerve (FN), lateral femoral cutaneous nerve (LFCN) and obturator nerve (ON). And it provides significant analgesia for the proximal femur. It can effectively reduce perioperative pain, obviously reduce postoperative opioids and shorten the length of hospital stay [1]. The key to the success of S-FICB is a sufficient volume of local anesthetic diffusion in the lacunae. A large volume of local anesthetic is often used to achieve perfect S-FICB effect in the clinic [2, 3]. However, most of the patients undergoing hip arthroplasty are elderly, whose multi-system function is decreased. Furthermore, they are complicated with cardiovascular and cerebrovascular diseases. A large volume of local anesthetic may increase the toxic reaction of local anesthetic and the incidence of complications in elderly patients. It is safer to use the minimum effective volume of local anesthetics. Therefore, in this study, the 95% effective volume of 0.33% ropivacaine for ultrasound-guided SFICB was measured by BCD, which provides a reference for the local anesthetic dosage of S-FICB in hip arthroplasty.

## Materials and methods

### Study design and participants

This study was approved by the Institutional Review Board of First Affiliated Hospital of Chongqing Medical University, Chongqing, China (approval number: 2015ZY102, approval date:26/07/2021). Letters of informed consent signed by patients were obtained. This study was conducted in accordance with the principles of the Declaration of Helsinki. The trial was registered at the Chinese Clinical Trial Registry (registration number: ChiCTR2100052214, registration date: 22/10/2021).

This study was conducted at First Affiliated Hospital of Chongqing Medical University from November 2021 to August 2022. This study examined patients aged 55 to 90, ASA II-III, BMI19.05 ~ 30.85 kg/m^2, and undergoing hip arthroplasty. The exclusion criteria were (1) apparent organ dysfunction such as heart, brain, lung, liver, and kidney, (2) coagulation dysfunction, (3) local skin infection at the puncture site, peripheral neuropathy, (4) Opioid abuse, chronic pain, unable to judge whether analgesia is effective or not, (5) Local anesthetic allergy, (6) patients with hypertension that drugs cannot control, and (7) Combined with psychiatric diseases and unable to cooperate.

### Study protocol

**Fig. 1:**
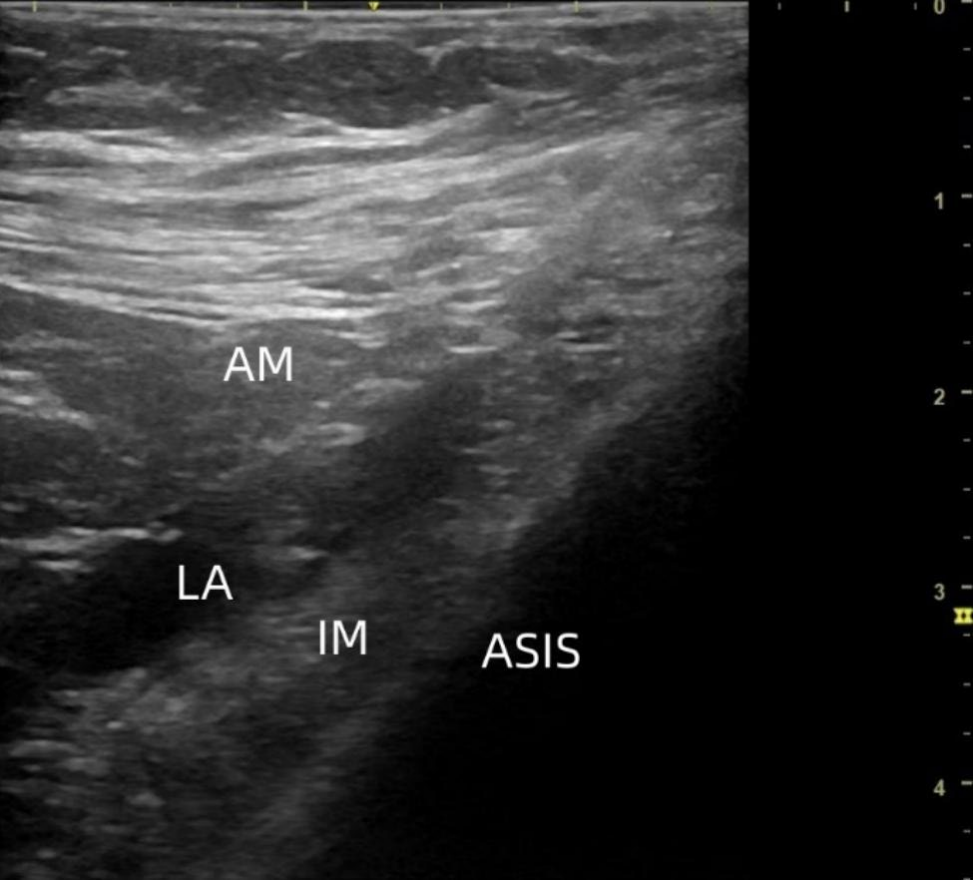
Ultrasound image of supra-inguinal fascia iliaca compartment block. ASIS Anterior superior iliac spine, IM iliacus muscle, AM abdominal muscles, LA local anesthetic

After entering the room, electrocardiography, pulse oximetry, and Invasive arterial blood pressure were applied. All blocks were performed by two anesthesiologists experienced in ultrasound-guided S-FICB with the patient in the supine position. After skin disinfection and draping, a high-frequency (13–6 MHz) linear ultrasound transducer (SonoSite M Turbo; Secma, Amsterdam, The Netherlands) was positioned longitudinally at the level of the anterior superior iliac spine (ASIS). The iliac muscle (IM) and fascia iliaca (FI) were identified by sliding the ultrasound transducer in a medial and caudal direction. To obtain a ‘bow-tie sign’ the transducer was rotated slightly so that the cranial end of the transducer points to the umbilicus and the caudal end pointed at the ASIS. The transducer was positioned in the parasagittal plane, and the deep circumflex iliac artery (DCIA) was identified, which is a vital ultrasound landmark. In this position, a needle was introduced at the transducer caudal edge. Using the in-plane approach, the fascia iliaca was penetrated and hydro-dissected, separating the fascia iliaca from the iliac muscle. The predetermined volume of 0.33% ropivacaine was injected (Fig. 1).

**Fig. 2:**
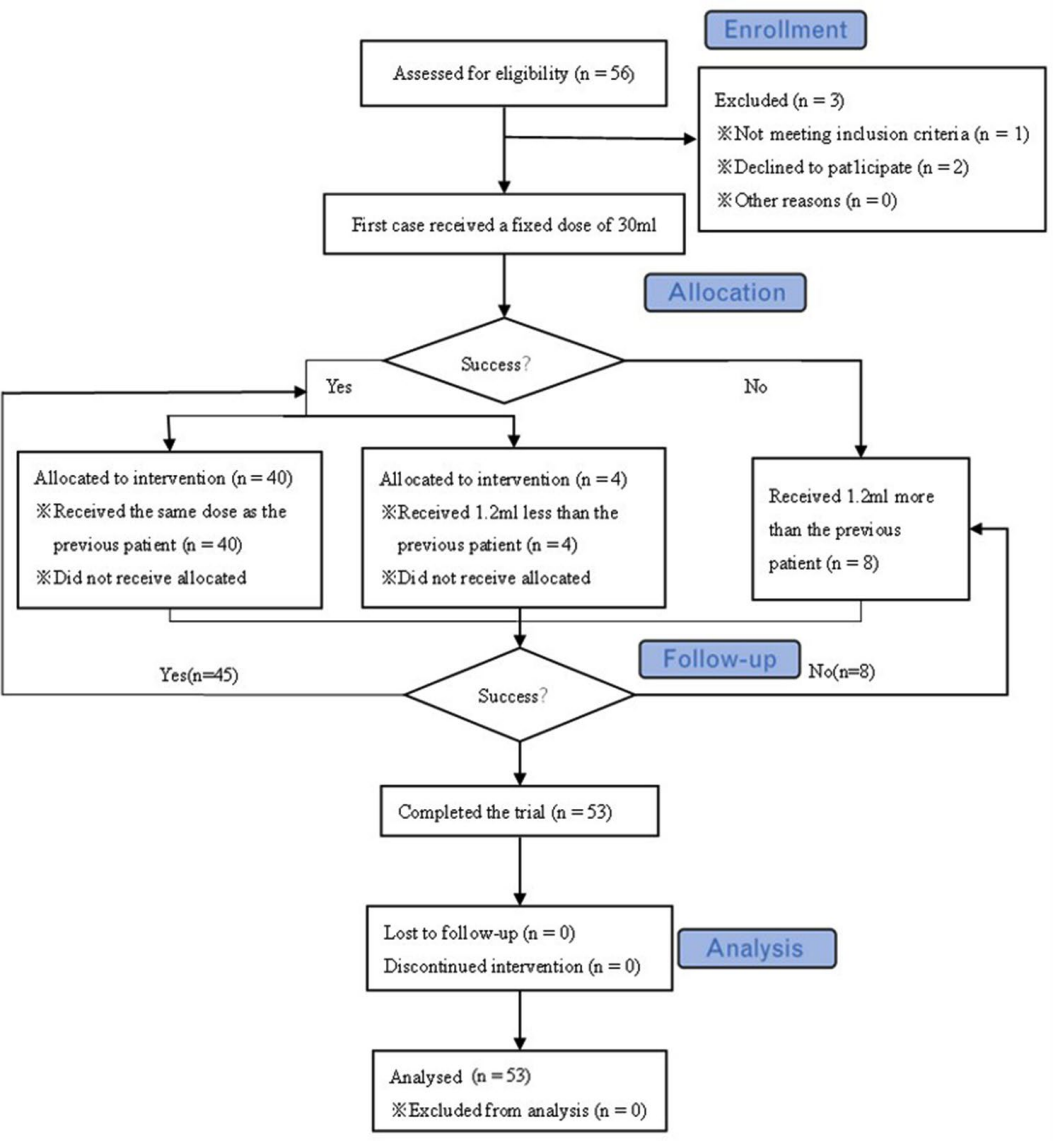
Flow diagram of study

Referring to the previous study [4, 5] and the pre-trial results, we set the initial volume at 30 ml and the interval volume at 1.2 ml [6, 7]. Block evaluation was performed 30 min after the injection, and the total volume administered to each patient depended on the response of the previous one. In case of failure, the next patient received a higher volume (defined as the previous volume with an increment of 1.2 mL). If the previous patient had a successful block, the next patient was randomized to a lower volume (defined as the previous volume with a decrement of 1.2 mL), with a probability of b = 0.05, or the same volume, with a probability of 1 − b = 0.95. The study was stopped when 45 successful blocks were achieved. Block evaluation was performed by a physician who was not involved in administering the S-FICB (Fig. 2). After evaluating the block effect, all patients were given midazolam(0.05 mg/kg), propofol(1 ~ 2 mg/kg), rocuronium(0.9 mg/kg) or vecuronium(0.1 mg/kg), and sufentanil(0.5 µg/kg). Endotracheal intubation and mechanical ventilation were performed after 5 min, control PETCO2 35 ~ 45mmHg. Sevoflurane 1% ~1.5% inhalation, maintain MAC value at 0.5, propofol 2 ~ 8 mg/kg/h, remifentanil 8 ~ 10ug/kg/h, add rocuronium or vecuronium and sufentanil as needed, and maintain BIS at 40–55.

Experienced surgeons performed all operations. The surgical approach was posterior total hip arthroplasty (Moore approach). The patient was in a lateral decubitus position, and the fixation was placed at the sacrum and pubic symphysis. And during the operation, the pelvis and trunk are perpendicular to the operating table, which is parallel to the floor. The incision ran from the distal side 10 cm of the posterior superior iliac spine to the posterior edge of the greater trochanter, parallel to the gluteus maximus muscle fibers. And then, it extended 10 ~ 13 cm distally along the femoral shaft.

All patients were sent to the resuscitation room after the operation. The tracheal tube was extubated after spontaneous breathing recovery, consciousness recovery, and muscle strength recovery. Send patients back to the ward when the steward score exceeds 4.

### Outcome measurements

This study evaluates the effect of the femoral nerve and lateral femoral cutaneous nerve to determine whether S-FICB is effective. Measurements of supra-inguinal fascia iliaca compartment blockade were carried out every 5 min until 30 min by a single, blinded observer. The sensation of pinprick reflects the effect of S-FICB. Sensory blockade of the FN was assessed on the anterior thigh; that of the LFCN was assessed on the lateral thigh. Compared with that before the S-FICB, it was grade 0 if the pain was no change, grade 1 if the pain decreased, and grade 2 if the pain disappeared completely. 30 min after the injection, the sensory blockade of the above two nerves reached grade 2, which can be judged that the block is successful. Otherwise, the block failed.

The muscle strength of the quadriceps femoris was measured and recorded before S-FICB and 30 min after S-FICB. The muscle strength of the quadriceps femoris was measured by the unarmed muscle strength classification method. Level 0: complete paralysis; Level 1: weak muscle contraction, but cannot cause joint contraction; Level 2: cannot resist gravity, can complete the movement of the whole joint; Level 3: can resist gravity but not resistance, can complete the movement of the whole joint; Level 4: can resist gravity and weak resistance, can complete the movement of the whole joint; Level 5: can resist gravity and strong resistance, can complete the movement of the whole joint. The complications such as puncture site bleeding, hematoma, local anesthetic poisoning, puncture needle into blood vessels, and hypoxemia were recorded.

### Statistical analysis

SPSS 25.0 software package and GraphPad Prism 5.00 were used for statistical analysis. Continuous data are expressed as the mean ± standard deviation (x ± SD) or median (interquartile range), and count data are expressed as the number of cases or a percentage. The difference between the two groups for non-normally distributed continuous variables was evaluated using Wilcoxon-Mann-Whitney *U* tests. Probit regression analysis was performed on LA volume data to calculate the EV50 and EV95 and their 95% CIs [8, 9]. p values lower than 0.05 were considered significant.

## Results

In total, 56 patients were enrolled in the study. Three patients were excluded from the study due to the exclusion criteria described above. The consort flow chart is shown in Fig. 2. Table 1 demonstrates the baseline characteristics of the 53 patients in this study. Thirty-one patients in the study were non-fracture patients. Quadriceps muscle strength decreased in only two patients. Moreover, they both received 34.8ml of ropivacaine for S-FICB (Table 2). Twenty-two patients had hip fractures. There were 3 patients (14%) with failed blocks, whose pain grade was grade 1. And there were 19 patients (86%) with successful blocks. However, all fracture patients experienced less pain after S-FICB.

**Table 1:** Demographic data of Study Participants, n = 53

Characteristics	n or mean ± SD
Sex (male/female)	20/33
ASA(II/III)	24/29
Age (year)	70±10
Height (cm)	159±8
Weight (kg)	58±10
BMI(kg/m^2^)	23±3
Operation duration (min)	60±21

**Table 2:** Muscle strength changes in non-fracture patients

Non-fracture patients	Total	Decreased	Decreased /Total
Success	26	2	2/26
Failed	5	0	0/5
Total	31	2	2/31

**Fig. 3:**
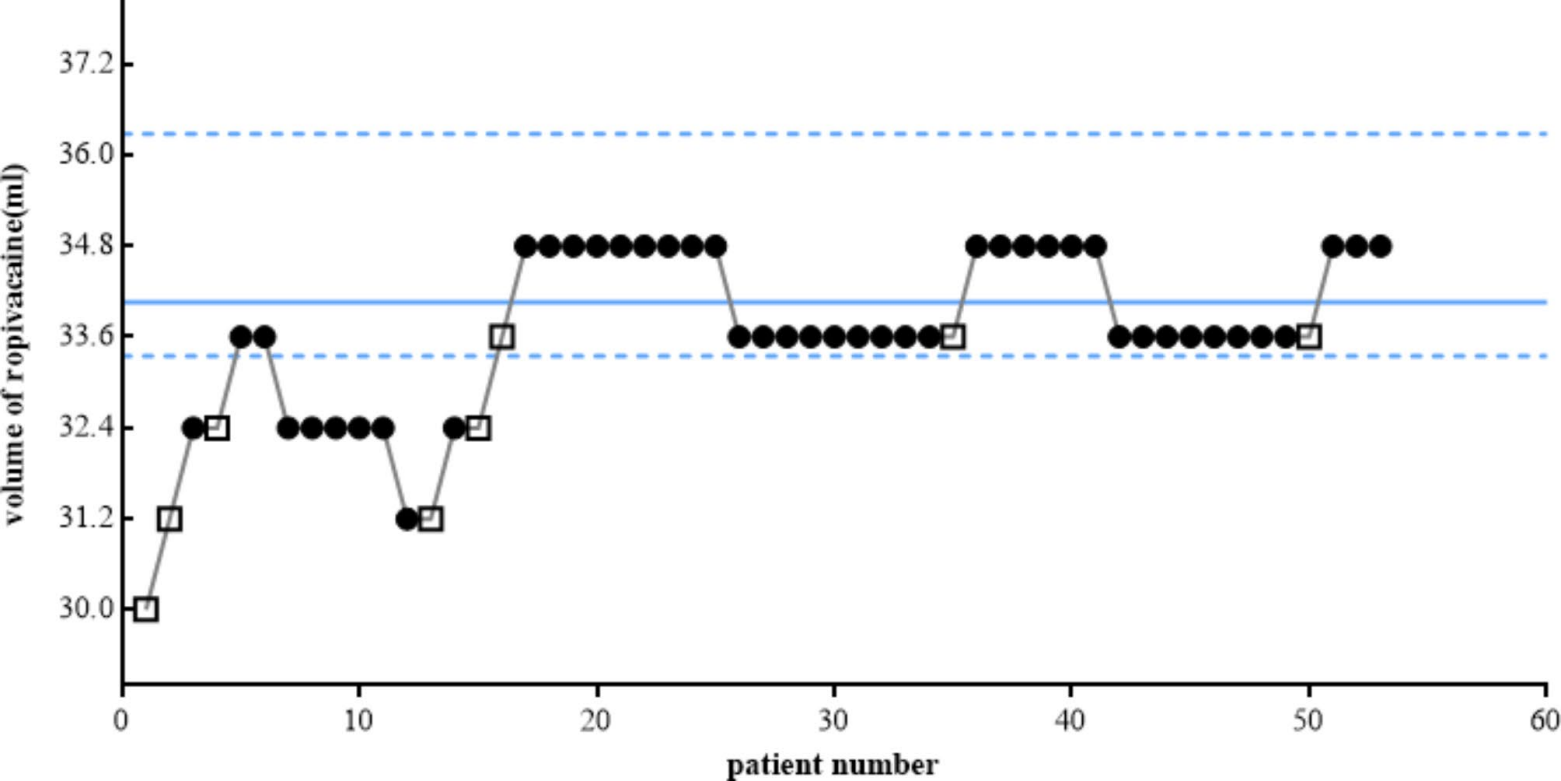
The up-and-down sequence of volumes of ropivacaine 0.33% required for ultrasound supra-inguinal fascia iliaca compartment block to produce effective femoral nerve block and lateral cutaneous nerve block. □ failed block, • successful block. The minimum effective volume 95 is represented as a continuous line and the 95% confidence interval as dashed lines

45 (85%) patients were blocked successfully. The onset time of the femoral nerve block and lateral femoral cutaneous nerve block were 15.0(2.5) min and 15.0(5.0) min, respectively. There was no significant difference between FN and FLCN in the block onset time(Z=0.53, p>0.05). 30 min later, the block effect of FN and LFCN was consistent in all patients. The EV50 of 0.33% ropivacaine is 31.75ml (95%CI, 29.16 ~ 32.50) and EV95 is 34.06ml (95%CI, 33.35 ~ 36.28). The sequence of positive and negative blocks in the evaluated patients is shown in Fig. 3.

All patients had no complications such as puncture site bleeding, hematoma, local anesthetic poisoning, puncture needle into blood vessels, and hypoxemia.

## Discussion

FICB is a technique of injecting the local anesthetic into a triangular space between the FI and the edge of the IM, which can effectively block the FN, LFCN, ON, and genitofemoral nerve (GFN). The block of FN and LFCN helps relieve the pain of hip arthroplasty. Depending on the puncture point choice, FICB has various operation methods [10–12]. The improved S-FICB was used in this study. Compared with the traditional FICB, this method can make the local anesthetic spread better in a cranial direction and block the FN, LFCN, and ON more completely [13]. It can also provide more effective analgesia and increase patients’ athletic stamina after the operation [4]. Hence, patients can get out of bed early. It has been widely recognized in clinical work.

The up-and-down method is currently recognized as a classical method for determining the 50% effective dose or concentration of drugs. The advantage is that it requires a small number of patients and can save many samples. It can also reflect the situation of any point in the dose-response curve [2, 14]. As a result, it is also widely used by Anesthesiologists. Some studies have used this method to determine the effective volume of ultrasound-guided FICB. However, most studies have focused on determining EV50 [5], relying on Dixon’s up-and-down method and then using Logistic regression or Probit regression to infer a higher quantile (e.g., EV95). However, this inference is not accurate. In contrast, the BCD used in our study is an improvement and upgrade of Dixon’s up-and-down method, which can directly study effective volume in any quantile. Hence, the determination results are more accurate. In this study, The EV95 of 0.33% ropivacaine for FICB was 34.06ml. Kantakam et al. [15] also used BCD to determine the EV90 of FICB, but the subjects were adult cadavers. It was a cadaver study in which dyes were injected into the FIC during the implementation of S-FICB. However, the diffusion of dyes and local anesthetics is different because of their intrinsic viscosity and density [16]. And some studies have shown that passive muscle movement in vivo can promote the diffusion of the local anesthetic to a cranial direction in the FIC [17]. Therefore, our study takes clinical patients as the subjects, and the results have more clinical reference significance. The volume of EV95 evaluated in our study is lower than that of 40ml commonly used in hip surgery [18, 19]. The EV95 not only meets the needs of analgesia during operation but also saves the dosage of local anesthetic. The risk of insufficient or ineffective analgesia due to insufficient volume of LA is reduced.

It is worth noting that in our study, the muscle strength of quadriceps femoris before S-FICB and 30 min after S-FICB in non-fracture patients was also included in the observation index. The results showed that only 2 of the 31 subjects’ muscle strength decreased at 30 min after S-FICB. And they both received 34.8ml of local anesthetic. The volume of 0.33% ropivacaine used in our study has little inhibition on lower limb muscle strength in patients undergoing hip arthroplasty. And this is positive for patients to participate in functional exercise, get out of bed as soon as possible and even prevent postoperative complications such as deep venous thrombosis.

This study also has some limitations. First, 0.3% ~ 0.5% ropivacaine is the recommended concentration range for peripheral nerve block. Single-point FICB often requires a larger volume of the local anesthetic to achieve a better blocking effect. And most of the patients included in this study are elderly patients. They are more sensitive to local anesthetics due to changes in their pathophysiology. Therefore, this study chose a lower concentration of ropivacaine. Considering the convenience of clinical operation, we finally chose 0.33% ropivacaine. The blocking effect of other concentrations of ropivacaine in different crowds needs further study. Second, the efficacy of the ON block in analgesia after hip arthroplasty is uncertain. Previous scholars [20, 21] proposed that the ON block cannot relieve pain after hip arthroplasty, and the ON block may not be necessary for postoperative analgesia [6]. Therefore, although some scholars have proved that S-FICB can effectively block the ON [22], we did not include the ON. Third, this study only observed patients’ pain within 30 min and did not continue to follow up on the postoperative situation, such as the duration of analgesia, pain scores during the first postoperative 24 h, and the total dose of morphine consumption during 24 h. These indexes can be used as a reference to recommend the volume of local anesthetics by comparing each volume subgroup.

## Conclusions

The 95% effective volume of 0.33% ropivacaine for ultrasound‑guided S-FICB was 34.06ml (95% CI, 33.35 ~ 36.28). In addition to volume, different local anesthetics, different concentrations, and whether adjuvants are added also affect nerve block efficacy. The results of the present study were obtained under the limited conditions of the study, and the dosages under other conditions require further study. These indicators can also be used as a reference to recommend the volume of local anesthetic.

## Data Availability

The datasets used and/or analyzed during the current study are available from the corresponding author on reasonable request.

## Abbreviations

S-FICB: supra-inguinal fascia iliaca compartment block
BCD: biased-coin design
EV50: 50% effective volume
EV95: 95% effective volume
LA: local anesthetic
FIC: fascia iliaca compartment
FN: femoral nerve
LFCN: lateral femoral cutaneous nerve
ON: obturator nerve
GFN: genitofemoral nerve
ASIS: anterior superior iliac spine
IM: iliac muscle
FI: fascia iliaca
DCIA: deep circumflex iliac artery
